# Combining Minimally Invasive Techniques in Managing a Frail Patient with Postpneumonectomy Bronchopleural Fistula

**DOI:** 10.1155/2021/5513136

**Published:** 2021-04-07

**Authors:** Kostas Kostopanagiotou, Dimitrios Filippiadis, Efthimios Bakas, Costas Thomas, Andreas Kostroglou, Santaitidis Elias, Tatiana Sidiropoulou, Sotirios Tsiodras, Periklis Tomos

**Affiliations:** ^1^Thoracic Surgery Department, Attikon University Hospital of Athens, Greece; ^2^2nd Radiology Department, Attikon University Hospital of Athens, Greece; ^3^4th Department of Internal Medicine, Attikon University Hospital of Athens, Greece; ^4^2nd Department of Anesthesiology, Attikon University Hospital of Athens, Greece; ^5^Department of Epidemiological Surveillance and Intervention, Hellenic Centre for Disease Control and Prevention, University of Athens, Greece

## Abstract

A postpneumonectomy bronchopleural fistula is a life-threatening complication requiring aggressive treatment and early repair. Reoperations are common due to initial treatment failure. Advanced bronchoscopic techniques are rapidly evolving, but permanent results are questionable. We report the minimally invasive management of a frail 79-year-old patient with postpneumonectomy fistula in respiratory failure due to repeated infections. Previous bronchoscopic closure attempts with fibrin failed. The multistep interdisciplinary management included airway surveillance by virtual bronchoscopy, percutaneous fibrin glue instillation under computed tomography, and awake thoracoscopic surgery to achieve temporary closure. This provided an acceptable long period of symptomatic and physical improvement. The bronchial stump failed again four months later, and the patient succumbed to pneumonia. Pneumonectomy has to be avoided unless strongly indicated. Complications are best managed with surgery for definite treatment. We emphasize our approach only when a patient declines surgery or is medically unfit as a temporary time-buying strategy in view of definite surgery in a high-volume center.

## 1. Introduction

The reported incidence for postpneumonectomy bronchopleural fistula (ppBPF) in a series of a high-volume center is 4.5%. This incidence decreases as experience grows as low as 1% [1]. This number can be higher across different practices in the world. Previous publications report incidences of up to 16% [2]. Thus, ppBPF is an accountable problem for respiratory physicians and thoracic surgeons to manage. Direct surgical repair has an indication in the early postoperative period often combined with an intercostal muscle flap. The situation is more complex in delayed diagnosis cases where pleural empyema and sepsis lead to borderline physiological reserves precluding patients for major thoracoplastic surgery [3]. The remaining options then are either an open window thoracostomy under local anesthetic or long-term tube drainage. At experimental level in experienced hands, various endobronchial interventions have been reported (stent, one-way emphysema valve, Amplatzer closure device, and biological sealants) [4–7]. However, these are lacking strong scientific evidence on safety or long-term results. Below, we describe how we set an interdisciplinary collaboration of interventional radiologists, respiratory and infectious disease physicians, a thoracic surgeon, and anesthetist to manage a borderline physiology patient declining surgery to provide a reasonably long symptom-free period until preparing for definite thoracoplasty.

## 2. Case Report

**Figure 1:**
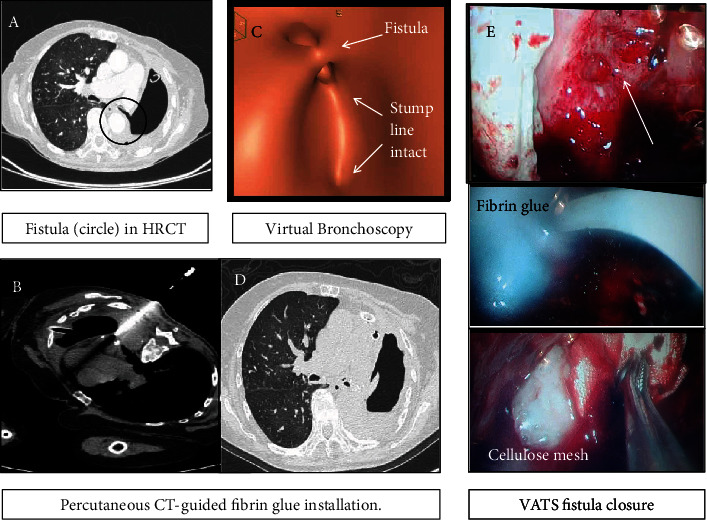
(a) The narrow fistula tract is the identifiable target for glue obliteration. (b) Percutaneous CT fibrin instillation. (c) Virtual bronchoscopy is useful for the estimation of the problem. (d) Airtight obliterated fistula after the water test. (e) Thoracoscopic images of a chronic bronchopleural fistula.

A cachectic female patient of 79 years of age with a chronic postpneumonectomy bronchopleural fistula presented at the emergency department with severe dyspnoea and cyanosis. This episode was the most severe of several recurrent respiratory infections. On examination, she had central cyanosis, tachypnoea of respiratory rate > 30/min, and pulse oximetry of 80% saturation on room air and 86% with a 4 L/min nasal cannula. Blood pressure was 88/55 mmHg, pulse was 105 bmp in sinus tachycardia, and core temperature is 38.3 degrees Celsius, and she was passing minimal urine. On auscultation, there was a silent left pneumonectomy side and bronchial wheeze on the right side. Arterial blood gases were acidotic and hypoxemic at pH 7.21 pO_2_ 58 mmHg and pCO_2_ 50 mmHg. Laboratory values were suggestive of infection with Hgb of 9.7 g/dL, white cell count of 17.8 K/*μ*L, and C-reactive protein of 184 mg/L. A long-term 32Fr left-sided intercostal chest drain was draining seropurulent fluid with a constant stream of bubbles. Her surgical history was notable. Previously fit and well, she underwent nine months earlier—in another institution—a left pneumonectomy through posterolateral thoracotomy for a benign insulin-producing hilar mass thought to be lung cancer. This hilar mass reportedly caused worsening bronchial obstruction, and it was not amenable to any other type of treatment or resection. A month after, the pneumonectomy became septic and bronchoscopy confirmed the presence of a bronchopleural fistula (ppBPF). At this time, she declined any form of surgery and it is unknown if she was aware of the possible complication risks at the time of consent. Unfortunately, much of the documentation was unavailable on presentation in our institution. At that time, she underwent repeated endobronchial instillations of fibrin glue at the initial institution and left for home with a percutaneous tube thoracostomy connected to a Heimlich valve and oral antibiotics for the initial weeks. At her decision, she declined further attempts to close the stump and decided only to attend a weekly outpatient clinic for topical care of her long-term chest drain. There was a steady decline in her overall condition with recurring respiratory tract infections due to aspiration pneumonias of the contralateral side until developing respiratory failure. That was the overall management until presentation to our emergency department for a first time review. At presentation, her CXR did show a reduced-volume postpneumonectomy left cavity with the drain in situ and shifting of the mediastinum to the left as expected. The right side had multiple parenchymal infiltrates indicating infection. On admission, we performed a contrast chest computed tomography scan, as there were no previous studies available for comparison on presentation. On grounds of differential diagnosis, we had to exclude the possibility of pulmonary embolism. The presence of a narrow opening at the bronchial stump site confirmed the diagnosis of ppBPF (Figure 1(a)). During admission, she received oxygen, nebulizers, and empiric intravenous antibiotics (tazobactam, 4.5 g QDS). A comprehensive set of sputum, blood, wound, and drain fluid microbiology cultures guided future antibiotic treatment. *Acinetobacter baumannii* and *Proteus mirabilis* were cultivated in the pleural fluid sensitive to colistin, piperacillin, and tazobactam. She remained on tazobactam since her inflammatory markers were improving. During the two-week long admission period, the patient improved. She remained afebrile with improved oxygen saturation of 88% on room air or 92% with a 2 L/min nasal cannula. She was discharged with home oxygen and nebulizers. The chest drain remained in situ with daily dressing changes by family members. Antibiotics continued for two more weeks at home by a private nurse. Nutritional supplements were prescribed to improve cachexia as her BMI on admission was 17. Additionally, a psychiatric specialist managed her postoperative depression symptoms with a serotonin-selective reuptake inhibitor. Thereafter, she attended our outpatient clinic every ten days for physical examination and inspection of her drain, which was connected to a Heimlich valve.

### 2.1. Planning the Bronchopleural Fistula Closure

After symptomatic improvement over the next two weeks, there was an extended discussion with the patient and family regarding surgery, expectations, and prognosis. We proposed an open window thoracostomy under local anesthesia. A vacuum therapy device in the stoma would complete our treatment plan. The patient declined general anesthesia, surgery, and bronchoscopy and insisted on noninvasive diagnostics or treatment. In order to investigate the morphology of the bronchial tree, we obtained an updated noncontrast high-resolution computed tomography scan. Then, we performed virtual bronchoscopy (VB) by digitally reconstructing the bronchial tree. There it was obvious that the stump was insufficient at the superior edge (Figure 1(c)). Although VB is less descriptive in detail than conventional fiberoptic bronchoscopy, it gives a useful estimation of the underlying problem. The partially open stump led to a fistulous tract (2 centimeters in length and 3 millimeters wide) between the stapled left hilum. All of which were covered in thick inflamed tissue. The first intervention to attempt ppBPF closure was a CT-guided percutaneous instillation of 10 mL of fibrin-based biological glue under local anesthesia with minimal pain (Figures 1(b) and 1(d)). This had an immediate effect stopping the air leak. The chest drain remained in place, and as a day-case procedure, the patient went home without any further treatment. After twenty days while at home, the stump opened again and air leak reappeared. On readmission, we proposed performing awake nonintubated video-assisted thoracoscopic (VATS) exploration. We use this technique regularly for minor VATS procedures (i.e., pleural biopsies) with minimal discomfort. The patient breathes spontaneously during the procedure. The standard regimen for mild sedation is intravenous dexmedetomidine at a titrable maintenance dose 0.5 *μ*g/kg/h. The supine position is more suitable over the lateral thoracotomy to avoid any debris moving through the fistula on the opposite lung. It was explained again that an open thoracostomy is the best option but the patient agreed only with the awake VATS option. We entered the chest through the existing drain hole by a 3 cm extension. The pleural cavity was debrided of minimal seropurulent fluid without septations and irrigated with 300 mL volume of normal saline. No extensive debridement was required in this thickened pleural cavity. Air bubbles were streaming in the ppBPF orifice. There was no visible bronchial stump to close with an automatic endoscopic stapler. Instead, a single “figure-of-eight” nylon suture was passed through the fibrous tissue at the opening site and tightened with a knot pusher. This appeared to stop the air leak during an underwater seal test. Then, we applied 10 mL BioGlue® (CRYOLIFE Inc., Kennesaw, GA, USA) and a cellulose mesh (Surgicel® Ethicon®, USA) on the site, and we recovered the dislodged previous BioGlue® cast placed initially (Figure 1(e)). There was no air leak at the end of the 25-minute long procedure and nothing more to offer surgically. No further irrigation was performed to avoid glue and mesh dislocation. A new 32Fr chest drain was secured in place and connected off-suction to a regular three-chamber canister. The procedure ended uneventfully with no dyspnea, pain, or discomfort. The postoperative hospital stay was two days long during which she remained afebrile and was discharged home with the chest drain in place and without antibiotics. No nebulizers or regular analgesics were necessary. The patient remained symptom and home oxygen free and attended the outpatient clinic every 10 days for chest drain care. The stump failed again after four months. At that time, the open window thoracostomy and vacuum therapy were agreed and scheduled; however, the patient died at home days later due to pneumonia.

## 3. Discussion

The fundamentals in managing this catastrophic complication are uniform in worldwide practice. An early pneumonectomy stump dehiscence requires early surgical repair usually with the use of an intercostal or other muscle flap. Delayed presentation cases require drainage and debridement of the cavity, prevention of contralateral lung aspiration, and antibiotics. Selectively to a group of fit patients, we offer an operation by a variety of approaches. These are either window thoracostomy commonly under local anesthesia, an omental or muscle flap fistula closure, thoracomyoplasty, or a combination of these techniques [3]. We suggested open thoracostomy and vacuum-sponge (VAC-type) device insertion in the stoma. We prefer this technique because it provides shorter healing time and longer intervals of wound dressing changes [8]. Creating a feeling of trust and focused interest on the patient's problem with minimal discomfort give us the time to improve a number of parameters from body mass to psychological strength and propose again surgery later in time. It is important to alter any feelings regarding the initial failure if we have to proceed to future reoperation. Alternatives to surgery are various interventional bronchoscopic techniques. However, their success depends strongly on equipment availability, budget, and local expertise. Strong evidence is lacking for use of stents in ppBPF and even more for endobronchial valves or Amplatzer closure devices [4–7]. Combinations of surgery and interventional bronchoscopy exist in the literature. Andreetti et al. successfully combined the insertion of a conical bronchial stent and subsequent open pleural packing with closure of the bronchial stump plus omentoplasty in 11 patients [9]. Our reported case presented a special challenge. Early at fistula presentation, she declined surgery. Then, repetitive interventional attempts by bronchoscopy—twice to our knowledge—were unsuccessful. Nine months later at the first presentation in our institution, her marginal physiology was prohibitive for general anesthesia. She declined a fenestration under local anesthetic. Thus, we opted for a percutaneous CT-guided biological glue instillation as the least invasive attempt with minimal discomfort and less-than-an-hour duration. We gain almost three useful weeks in which the dietician team improved her nutrition status with energy supplements and a psychiatrist progressively managed her depression. When the stump failed again, we opted for the awake thoracoscopic (VATS) debridement and exploration of the chest cavity under mild sedation. This is generally well accepted and even indicated in very ill patients [10]. The combination of single nylon suture, BioGlue®, and Surgicel® effectively controlled the air leak. This was a highly unlikely permanent measure. Irrigation of the cavity with antibiotics postoperatively was thought to be unnecessary as this chronic cavity had only thickened pleura without any aggressive infection. One may argue why not attempting this approach in the first place since awake VATS is free of the anesthesia risks. We assumed that if this frail patient improved within the initial weeks, we could explain and obtain consent to proceed straight to surgical repair. This would be a winning choice for her keeping in mind the nonmalignant primary pathology. Unfortunately, this did not happen; thus, the agreed thoracoscopic closure attempt was the only remaining option for that moment. Besides, the patient then had not consented for any thoracoplasty. The subsequent four-month freedom of respiratory symptoms ended before anything else was performed, but we learned important lessons. For chronic patients, any additional symptom-free time is of great importance. The presence of a supportive environment including dieticians, a psychiatrist, and experienced palliative nursing staff is crucial for improving the general status. The collaboration of surgeons, interventional radiologists, respiratory physicians, infectious disease experts, and a competent thoracic anesthesiologist in awake VATS was critical in designing a treatment plan. This buying-time strategy using minimally invasive techniques is useful in providing time to recover and plan more treatment options. It is critical to mention that any minimally invasive alternatives do not provide guaranteed results and there is no adequate published evidence on their side.

## 4. Conclusion

In this report, we describe the combination of minimally invasive diagnostics and interventions to act potentially as a bridge to definite treatment for marginal patients. Virtual bronchoscopy, percutaneous CT-guided fibrin glue instillation, and awake thoracoscopic surgery temporarily controlled the air leak and recurrent infections. We must keep in mind that pneumonectomy is a high-risk procedure for postoperative complications. Ideally, it should be avoided and whenever possible it should be performed in high-volume experienced centers. The severity of the potential complications—unless an emergency—should be well explained prior to surgery to avoid any future objections in complication management. Bronchoscopic interventions to manage bronchopleural fistulas carry no sufficient published evidence. A postpneumonectomy bronchopleural fistula is an indication for surgery by a variety of techniques. We advise meticulous debridement, stump coverage by a muscle flap, and vacuum therapy. The close collaboration between different specialty experts is mandatory especially in the event of failures.

## Data Availability

All relevant data have been included in the article.
